# Case report: Back pseudocyst of unknown origin

**DOI:** 10.1016/j.ijscr.2020.08.022

**Published:** 2020-08-29

**Authors:** Moustafa Moussally, Joanna Khalifeh, Imad Mokalled, Walid Faraj, Mohamad J. Khalife

**Affiliations:** Department of Surgery, American University of Beirut Medical Center, Beirut, Lebanon

**Keywords:** Pseudocyst, Soft tissue mass, Back tumor, Case report, Traumatic cyst

## Abstract

**Introduction:**

Soft tissue masses are fairly encountered clinical entities. Among the rare forms of soft tissues masses are non-pancreatic pseudocysts which are defined as encapsulated fluid collections not lined by epithelium. We are presenting a rare case of a pseudocyst of the back located within the paravertebral muscles at a tertiary care center.

**Case description:**

Patient presented with a progressively enlarging painless back mass. CT scan was done which showed a 10 × 10 cm cystic lesion along the paravertebral muscles of the back. Surgical excision of the mass was performed revealing an irregularly shaped multilocular cyst. Final pathology showed the lesion to be a pseudocyst.

**Discussion:**

Pseudocysts can arise in a variety of locations such as omentum, adrenal glands, spleen or lungs. The etiology of these pseudocysts is highly variable and can be related to trauma, inflammation, or surgery. The diagnosis of these entities requires proper imaging and histopathologic examination.

**Conclusion:**

Pseudocysts arise due to a myriad of conditions and their diagnosis remains a challenge. Our case comprises the first reported case of idiopathic pseudocyst of the back. Although rare, pseudocysts should be considered in the differential of unexplained masses.

## Highlights

• Pseudocysts are defined as encapsulated fluid collections not lined by epithelium.
• The etiology of pseudocysts is highly variable and can be related to trauma, inflammation, or surgery.
• Morel-Lavallee lesion also known as closed degloving injury or post traumatic pseudocyst usually arises secondary to trauma or shearing force.
• Soft tissue masses are frequently reported complaints while nonpancreatic pseudocysts remain rare entities.

## 1 Introduction

Soft tissue masses are common and regularly encountered clinical entities [1,2]. The etiologies of soft tissue masses are variable, and can be secondary to trauma, infection, malignancy and unknown causes [2]. The majority are benign in nature [2]. They present as painless progressively enlarging masses [2]. History and physical examination is of utmost importance in the diagnosis of those masses [2]. Patient’s age and the anatomical location of the mass can help in the differential diagnosis [1]. They could be cutaneous, subcutaneous, fascial or deep [1]. Among the rare forms of soft tissues masses are non-pancreatic pseudocysts [3]. These pseudocysts vary in location and symptomtology [3]. We are presenting a rare case of a pseudocyst of the back located within the paravertebral muscles at a tertiary care center. We explore the various possible diagnoses and perform a literature review of pseudocysts.

## 2 Case presentation

A 62-year-old non-smoker male patient presented to our hospital with a 6 months’ history of progressively enlarging mass along his back. It was first noticed 2 years ago upon palpation, with time it has gradually increased in size and became cosmetically noticeable. No history of pain, fevers, weight loss, or trauma.

**Fig. 1:**
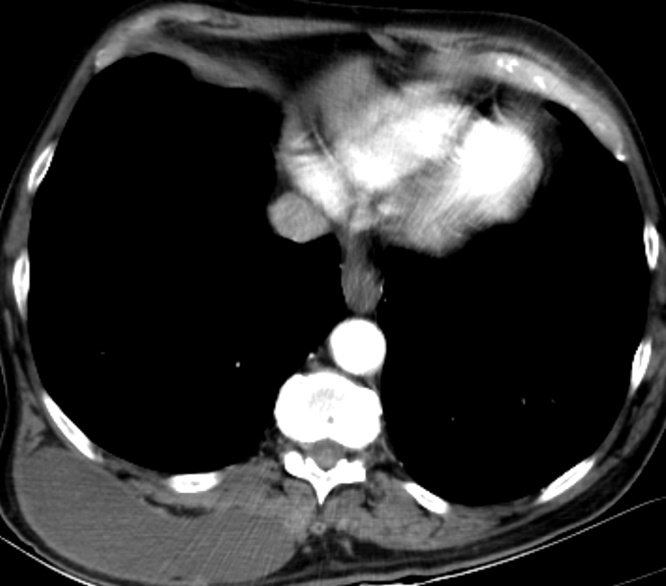
CT Scan of the Chest showing a 10 cm × 10 cm cystic lesion in the right thoracic region.

**Fig. 2:**
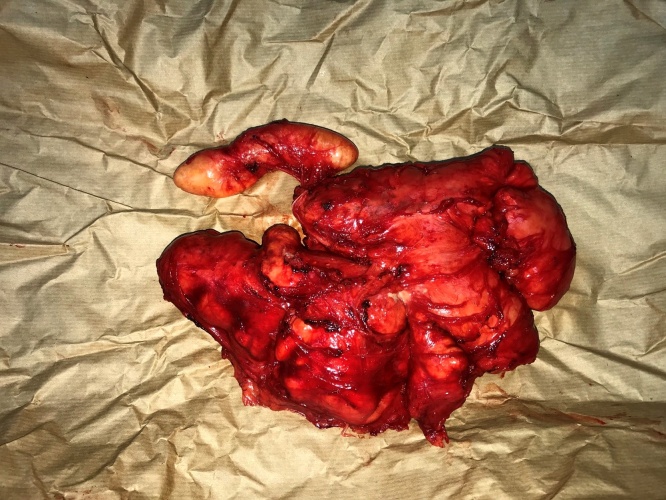
Gross examination of the excised specimen.

**Fig. 3:**
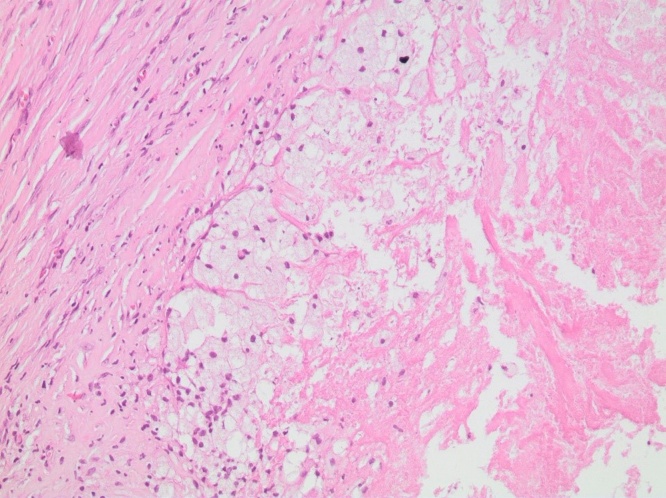
Hematoxylin and Eosin Stain of the tumor at 20× magnification showing clusters of macrophages with few mononuclear leukocytes.

Physical exam revealed a 10 cm soft, immobile, painless mass along the right paravertebral with no overlying discoloration of the skin. Computed Tomography (CT) Scan (Fig. 1) revealed a 10 × 10 cm cystic lesion along the paravertebral muscles of the back. Given the patient’s exposure to cattle and dogs as well as prolonged ingestion of raw food, the lesion was considered to be most likely hydatid disease of the back muscles. The decision was made to proceed with surgical excision by Dr. Walid Faraj and Dr. Mohamad J. Khalife both of whom are Hepatopancreaticobiliary surgeons with vast experience with hydatid disease. The patient was started on Albendazole 400 mg twice daily for one week prior to surgery. Intraoperatively, a vertical incision was made along the lesion. Dissection was done reaching the mass with care to avoid spillage of contents. The mass was then injected with scolicidal agent cetramide 1.5% to prevent dissemination. The mass surrounding attachments were lysed. The mass was dissected off its attachments to the underlying paravertebral muscles at its roots completely. A multilocular cyst showing outer adhesions was excised as one entity. On gross examination, the lesion looked irregular and contained beige yellowish creamy material (Fig. 2). The wall of the lesion was elastic in nature. The inner aspect of the lesion was focally beige and granular. On microscopic examination, the sections showed fibrous cystic wall adhering to the surrounding connective tissue. Furthermore, the inner aspect was covered by large clusters of macrophages having microvacuolated cystoplasm and mixed with few mononuclear leukocytes (Fig. 3). The mass was diagnosed as a pseudocyst formed around liquefied soft tissue, surrounded by fibrous capsule and histiocytic reaction.

## 3 Discussion

Pseudocysts are defined as fluid collections within a capsule not lined by epithelium [4,5]. Pseudocysts can arise in a variety of locations. They can be intraperitoneal such as omental pseudocysts, retroperitoneal such as pancreatic or adrenal pseudocysts, splenic or pleural [[3], [4], [5], [6], [7], [8], [9], [10]]. However, no cases of pseudocysts arising in the back have been reported in the literature. The majority of reported pseudocysts are iatrogenic in nature. They arise following incisional hernia repair with mesh, intraperitoneal dialysis catheter or ventriculo-peritoneal shunt surgeries [4,5]. The prevalence of pseudocysts following hernia repair using mesh has been reported to be 0.88% in some studies [4]. In addition, there has been a few reports of idiopathic omental pseudocysts [7].

Trauma comprises another cause of pseudocysts. Traumatic pulmonary pseudocysts arise following blunt chest trauma [10]. The formation of these pseudocysts is attributed to the persistence of an abscess or a hematoma [3]. Chronic Morel-Lavallee lesion (MLL) is a possible diagnosis for the case at hand. Morel-Lavallee lesion also known as closed degloving injury or post traumatic pseudocyst was described initially in 1853 by Dr. Maurice Morel-Lavallee [11,12]. It usually arises secondary to trauma or shearing force which subsequently results in the separation of subcutaneous tissue from the deep muscle fascia. This leads to the creation of a space which can be filled by blood, lymph, or necrotic fat [13]. While the majority of these lesions arise directly after injury, approximately one third of lesions might present gradually months to years after the inciting event [11,12]. When left untreated, these gradually growing lesions might often be confused with a soft tissue tumor [14]. Delayed diagnosis of MLL leads to the formation of granulation tissue which create a pseudo-capsule. This hinders the absorption of the contents of the lesion [[11], [12], [13], [14]]. In such cases, percutaneous drainage is futile and surgical intervention is necessary. These lesions can be detected and diagnosed by Ultrasonography, Computed Tomography or Magnetic Resonance Imaging [12]. MRI remains the ideal imaging modality for the diagnosis of MLL [11,15]. The presence of pseudo-capsule can be identified on MRI; this is essential information that aids in determining the next step in management [12]. In our patient, the symptomatology, chronicity and smoothly marginated gross appearance of the lesion favors MLL. Although the diagnosis of MLL is still a possibility, the absence of fluid collection within the lesions renders this diagnosis unlikely.

In addition to traumatic causes of enlarging mass on the back, the possibility of soft tissue tumor should be considered. Soft tissue tumors are highly variable and often require imaging as an initial step [2]. Imaging modalities such as US, CT, and MRI aid in narrowing the differential diagnosis [1,2]. Possible etiologies include mesenchymal tumors such as lipomas, sarcomas, angiomas, or neurofibromas [1]. Moreover, myxomas and melanomas comprise further possible causes of back tumors. Cases of paravertebral clear cell and myxoid sarcomas as well as benign tumors such as elastofibromas have been reported in the literature to be present in the paravertebral area [[16], [17], [18]]. Although our patient did not have any constitutional symptoms, this does not eliminate the possibility of malignancy as the presence of these symptoms is rare [11]. The results of the CT scan were not suggestive of any mesenchymal tumor. Moreover, the histopathological examination of the specimen did not reveal any neoplasm. Our microscopic evaluation revealed the mass to be a pseudocyst surrounding liquefied soft tissue.

## 4 Conclusion

Our patient represents an unusual case of a massive back pseudocyst. Whilst soft tissue masses are frequently reported complains, nonpancreatic pseudocysts remain rare entities. The etiology of these pseudocysts is variable and can be related to trauma, inflammation, or surgery. Our case comprises the first reported case of an idiopathic pseudocyst of the back. However, pseudocysts should be considered in the differential of such presentations. Identifying pseudocyts is a challenge. Thus, imaging is a valuable tool in diagnosing these lesions. Furthermore, histopathological examination is essential for tissue diagnosis [19].

## Declaration of Competing Interest

None.

## Funding

None.

## Ethical approval

Exemption from ethical approval was provided.

## Consent

All patient identifiers were removed. Patient consented for the publication of this case report.

## Author contribution

WF & MK did the surgery. MM, JK, and IM did the literature review and wrote the manuscript. WF & MK edited the manuscript.

## Registration of research studies

1. Name of the registry: NA.
2. Unique identifying number or registration ID: NA.
3. Hyperlink to your specific registration (must be publicly accessible and will be checked): NA.

## Guarantor

Mohamad J. Khalife, M.D, FRCP, FACS, FRCS.

## Provenance and peer review

Not commissioned, externally peer-reviewed.
